# 18-Fluorodeoxyglucose positron emission tomography/computed tomography for large vessel vasculitis in clinical practice

**DOI:** 10.3389/fmed.2023.1103752

**Published:** 2023-01-19

**Authors:** Kladoum Nassarmadji, Anthony Vanjak, Venceslas Bourdin, Karine Champion, Ruxandra Burlacu, Stéphane Mouly, Damien Sène, Cloé Comarmond

**Affiliations:** Department of Internal Medicine and Clinical Immunology, Lariboisière Hospital, Université Paris Cité, Paris, France

**Keywords:** 2-[18F]FDG-PET/CT, giant cell arteritis, takayasu arteritis, large vessel vasculitis, large vessel arteritis

## Abstract

Diagnosis, prognostic assessment, and monitoring disease activity in patients with large vessel vasculitis (LVV) can be challenging. Early recognition of LVV and treatment adaptation is essential because vascular complications (aneurysm, dilatations, ischemic complications) or treatment related side effects can occur frequently in these patients. 18-fluorodeoxyglucose positron emission tomography/computed tomography (2-[18F]FDG-PET/CT) is increasingly used to diagnose, follow, and evaluate treatment response in LVV. In this review, we aimed to summarize the current evidence on the value of 2-[18F]FDG-PET/CT for diagnosis, follow, and treatment monitoring in LVV.

## Introduction

Giant cell arteritis (GCA) and Takayasu arteritis (TA) are two vasculitis predominantly affecting large vessels: aorta and its major branches (1). They differ by their clinical presentation, prognosis, and treatment. Imaging modalities such as ultrasound (US), computed tomography (CT) and 18-Fluorodeoxyglucose positron emission tomography (2-[18F]FDG-PET/CT) are more frequently used, have replaced angiography and have modified management of these diseases (2).

18-Fluorodeoxyglucose positron emission tomography/computed tomography is a functional imaging modality of fundamental utility in oncology that has progressively been used in rheumatic diseases. Indeed, 2-[18F]FDG-PET/CT has shown in preclinical models the ability to detect glucose intake in inflammatory and endothelial cells (3, 4). In this review, we aim to illustrate the usefulness of 2-[18F]FDG-PET/CT in management of LVV.

## 2-[18F]FDG-PET/CT and giant cell arteritis

### 2-[18F]FDG-PET/CT in GCA diagnosis

Giant cell arteritis is the most frequent large vessel vasculitis affecting patients older than 50 years with a prevalence of 9/100,000 in a prospective study of a German population and up to 25/100,000 in patients older than 50 years (5). Diagnosis of GCA is based on the presence of clinical signs of vasculitis, proof of vessel inflammation, eliminating alternate diagnosis and dramatic response to steroids in patients older than 50 years.

Giant cell arteritis encompass cranial and extracranial manifestations. Constitutional symptoms and elevated inflammatory markers are present in >90% of cases and patients may present with fever of unknown origin as the initial symptom in 15% of cases (6, 7). Cranial manifestations such as headaches may present in two third of patients (8). The most severe acute complication, visual loss, is described in around 20% of cases but this has been reduced with early recognition of disease and usage of temporal artery ultrasound (9, 10). Pseudomyalgia rheumatica (PMR) is the most common extra cranial manifestation in GCA and occur in 45–50% of GCA patients (11). Clinical manifestations of large vessel involvement (limb claudication, thoracic pain) may develop in one fifth of GCA patients (12).

Temporal artery biopsy (TAB) was initially recommended in every case of suspected GCA and was considered the gold standard (13). However, results are delayed and biopsy may be negative in up to 42% of patients with predominantly large vessel GCA (LV GCA) (12). Temporal artery ultrasound has shown very good performance with a pooled sensitivity of 77% and a pooled specificity of 96% as compared with the clinical diagnosis of GCA (2). It is also cost effective compared to TAB but remains limited for the exploration of aorta and visceral arteries (14). Thus, it is the first line recommended imaging technique for suspected predominantly cranial GCA (2). Nevertheless, TAB remains strongly recommended over imaging in ACR 2021 guidelines (15). Recently, the 2022 American College of Rheumatology/EULAR GCA classification criteria emphasized the use of 2-[18F]FDG-PET/CT, as well as other investigative methods: Ultrasound, MRI, for use in clinical practice (16). PET, MRI, and CT are equally proposed to detect large vessel inflammation in GCA in recommendations from different scientific societies: ACR, EULAR, the British Society for Rheumatology and the French study Group for Large Vessel Vasculitis (2, 15, 17, 18).

18-Fluorodeoxyglucose positron emission tomography/computed tomography has overall good performance for the diagnosis of GCA. Specific patterns of PET/CT uptake show that patients with GCA and positive 2-[18F]FDG-PET/CT are more likely to have a diffuse disease with thoracic and abdominal aorta, bilateral subclavian and axillary arteries involvement (19). Ascending aorta is the most affected zone (72%) followed by the brachiocephalic trunk (62%), aortic arch (60%), and descending aorta (60%) (20).

Blockmans et al. (21) have compared PET versus TAB performance and found a sensitivity of 77% and a specificity of 66%. Subsequently, three meta-analysis including studies of GCA patients comparing PET alone or with CT vs. different gold standard (clinical diagnosis or TAB) found sensitivity of 80–89% and specificity of 89–98% (22–24). The main limitations of these meta-analysis are the inclusion of predominantly retrospective studies and the usage of different reference standard between included studies. More recently, a longitudinal prospective study comparing 2-[18F]FDG-PET/CT with clinical diagnosis at 6 months found a sensitivity of 67%, a specificity of 100%, a negative predictive value of 64% and a positive predictive value of 100% (25).

18-Fluorodeoxyglucose positron emission tomography/computed tomography is also a useful imaging technique to assess large vessel involvement in patients with suspected GCA and negative TAB. In a retrospective study of 63 patients with suspected GCA and negative TAB, large vessel involvement with 2-[18F]FDG-PET/CT was observed in 14 patients (22%). The final diagnosis of GCA was based on the presence of clinical symptoms, laboratory results, imaging data compatible with GCA, and good response to corticosteroid therapy (26).

**FIGURE 1:**
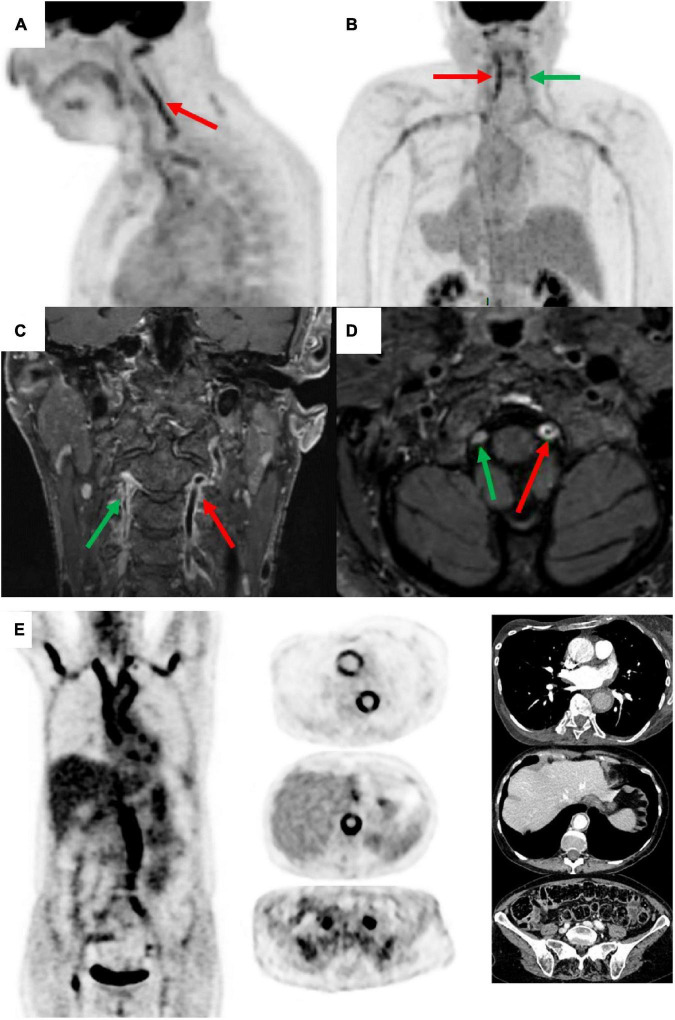
Positron emission tomography (PET) and magnetic resonance angiography (MRA) in a patient with giant cell arteritis (GCA) (man of 73 years old, CRP 17 mg/L, TAB positive). PET shows an inflammatory pattern with clear uptake (>liver uptake, grade 3) in vertebral arteries (left = red arrow and right = green arrow), sub-clavicular arteries, aortic arch, and thoracic aorta [panels **(A,B)** posterior image]. MRA shows vertebral arterial wall thickening, occlusion, and parietal enhancement [panels **(C,D)**]. PET and compute tomography angiography (CTA) illustrating aortitis at diagnosis in GCA patient, woman of 64 years old, CRP 84 mg/L, TAB negative [panel **(E)**].

Moreover, new generations of 2-[18F]FDG-PET/CT provide improved image resolution and can detect arteritis in smaller cranial arteries (temporal, maxillary, vertebral and occipital arteries) (Figure 1). Diagnosis of cranial artery inflammation with head, neck and chest PET/CT before or within 72 h after glucocorticoids intake showed a sensitivity of 82–92% and a specificity of 85–100% for diagnosis of GCA (27–29).

Finally, 2-[18F]FDG-PET/CT can be helpful in patients presenting with extracranial manifestation of GCA. In patients presenting with fever of unknown origin (FUO), abnormal 2-[18F]FDG-PET/CT increase the diagnosis rate to 83% among whom one-third have inflammatory diseases, such as vasculitis (30). In patients with suspicion of PMR, prospective studies using 2-[18F]FDG-PET/CT revealed the presence of LVV involvement in 31–60% of patients (31, 32).

The main differential diagnosis of FDG vessel uptake in vasculitis is atherosclerosis. Based on qualitative and quantitative vascular 18 FDG uptake, vascular site involved and disappearance upon steroid treatment, some differences can be noted (33): In atherosclerotic disease, uptake is usually low to moderate (Grade 0–1), has a patchy pattern and is predominantly located on iliofemoral sites and aortic bifurcations. In vasculitis however, FDG uptake is usually intense: a grade 3 uptake is found in aortitis only and semi quantitative analysis of FDG uptake are significantly higher in aortitis compared to atherosclerotic disease (mean SUVm 4.6 vs. 2.7) (34). Furthermore, FDG uptake in aortitis has a concentric, smooth linear pattern and may affect whole aorta. Also, CT images show non-concentric calcifications in atherosclerotic disease versus circumferential aortic wall thickness of more than 2–3 mm in vasculitis (35; Table 1).

**TABLE 1:** Differences in the pattern of fluorodeoxyglucose (FDG) uptake between vasculitis and atherosclerosis.

	Large-vessel vasculitis		Atherosclerosis
**Intensity of FDG uptake**	**Grade 2–3**		**Grade 0–1**
Pattern of uptake	Concentric, smooth, and linear		Patchy
Sites	GCA: Diffuse, disease, ascending aorta >brachiocephalic trunk and vertebral arteries >aortic arch = descending aorta	TAK: Axillar, subclavian, and common carotid arteries, abdominal aorta and renal, mesenteric arteries	Iliofemoral arteries, aortic bifurcations.
Calcifications on CT images	No		Yes
Response to steroids	Usually, disappear		Doesn’t change

Source Liozon et al. (19), Slart et al. (35), Gribbons et al. (79).

### 2-[18F]FDG-PET/CT in GCA prognosis

The prognosis in GCA is dominated by irreversible vision during short term course and vascular complications (dilatation, dissection, aneurysm, atherosclerosis) during long term follow-up (36).

Positron emission tomography/computed tomography is not adequate to evaluate the risk of vision loss because if ocular involvement is suspected, glucocorticoids must be started immediately and no imaging should delay the treatment (2). Moreover, ciliary arteries and central retinal arteries which are involved in ocular retinal damages are too small to be evaluated by PET/CT. Patients with GCA have a 2-fold increased risk of aortic aneurysm than control in a large UK cohort (37). Approximatively 20% of patients with GCA may develop aortic structural damage (aneurysm, dissection) (38, 39), mainly after 5 years from diagnosis (40).

Some risk factors for aortic damage in GCA have been identified and include male sex, smoking, hypertension and diabetes (37).

In two prospective studies by Blockmans et al. (31) and Galli et al. (41) assessing FDG uptake at diagnosis and during follow up, respectively up to 6 months and with a mean 97 months, vascular uptake at diagnosis did not predict subsequent relapse. However, an increased FDG uptake in the aorta at the diagnosis of GCA was associated with development of thoracic aorta dilatation (42) and in a prospective study including both GCA and TAK, future clinical relapses were more frequent in patients with a high PETVAS (≥20) than in patients with a low PETVAS (55 vs. 11%; *P* = 0.03) over a median follow-up of 15 months (43). More recently, the presence of FDG-PET activity at baseline in arterial territories of patients with LVV (TA or GCA) preceded angiographic progression and change (44). An arterial territory with baseline PET activity had 20 times increased odds for angiographic change compared to a paired arterial territory without PET activity. Concomitant edema and wall thickness further increased risk for angiographic change (44).

### 2-[18F]FDG-PET/CT in monitoring GCA activity

Therapeutic options for GCA comprise glucocorticoids (GC), tocilizumab (TCZ) and methotrexate (MTX). The optimal length of therapy is not well-known but treatment is usually maintained at least 2 years (15, 45). Indeed, relapses have been reported in around 30% of cases in prospective studies, mainly during the first 2 years following diagnosis (46, 47).

18-Fluorodeoxyglucose positron emission tomography/computed tomography in GCA can detect active aortitis and localize inflammation for extra-cranial arterial territories and peripheral arthritis (bilateral shoulder/hip pain and morning stiffness compatible with polymyalgia rheumatica–PMR) (2, 7). An activity score has been proposed to compare uptake evolution and is based on the sum of visual scores in different arterial regions: the Total Vascular Score (TVS). This visual score uses a standardized 0–3 grading system: 0 = no uptake (≤mediastinum); 1 = low-grade uptake (<liver); 2 = intermediate-grade uptake (=liver), 3 = high-grade uptake (>liver). Grade 2 is considered possibly indicative and grade 3 is considered positive for active LVV. The total score can be determined at seven different vascular regions (thoracic aorta, abdominal aorta, subclavian arteries, axillary arteries, carotid arteries, iliac arteries, and femoral arteries) and ranges from 0 to 21 (35). An increased number of vascular region can be chosen in a similar score: PET vascular activity score (PETVAS) by including four segments of the aorta (ascending, arch, descending thoracic, and abdominal) and five branch arteries (carotids, brachiocephalic trunk, subclavian/axillary arteries) with a maximum score of 27 (43).

In a prospective study of 29 patients with biopsy proven GCA and initially positive 2-[18F]FDG-PET/CT, TVS decreased from baseline to 3 months after treatment but remained unchanged at 6 months (31). Furthermore, there was no significant correlation between PET activity and clinical score (BVAS) or biological markers of activity (CRP, ESR) in patients with vascular complications or persistent inflammatory markers despite treatment (48). The persistence of FDG uptake despite clinical and biological remission is poorly understood (vascular remodeling vs. persistent mural inflammation) and its role in further vessel damage is unknown and is among the future research agenda (2).

The role of 2-[18F]FDG-PET/CT for treatment monitoring in LVV has been recently reviewed by van der Geest et al. (49). Longitudinal studies showed a decrease of baseline arterial FDG uptake after treatment induced remission. Investigation of early changes upon glucocorticoid treatment showed the persistence of FDG uptake after 3 days but its disappearance in 64% of cases after 10 days (50). The meta-analysis of four cross-sectional showed a moderate diagnostic accuracy for detecting active disease with a pooled sensitivity of 77% (95% CI 57–90%) and specificity of 71% [95% CI (47–87%)] (49). In a subsequent study comparing treatment effect on vascular inflammation, MTX and TCZ were associated with a higher decreased PETVAS than corticosteroids alone (51). The PET vascular activity score is useful to differentiate active and inactive disease and to predict relapse. However, PET/CT seems less accurate to evaluate clinically active disease in GCA compared to TAK probably explained by a younger age and less atherosclerosis in TAK, and a spectrum of cranial and articular clinical manifestations less frequently the expression of the LVV inflammation in GCA population.

There are no studies available using 2-[18F]FDG-PET/CT alone to guide treatment adaptation. 2-[18F]FDG-PET/CT provides information about vascular inflammation that is complementary from clinical assessment in LVV. A prospective imaging study in patients with GCA treated with tocilizumab shows that 2-[18F]FDG-PET/CT activity is significantly reduced in response to treatment with tocilizumab and repeat 2-[18F]FDG-PET/CT after tocilizumab discontinuation reveal worsening vascular PET activity in most patients (52). Therefore, treatment adaptation is guided by multimodal assessment with clinical, biological and imaging parameters. The 2-[18F]FDG-PET/CT place remains to be specified but 2-[18F]FDG-PET/CT persistent uptake despite clinical remission could be associated with future clinical relapse.

### 2-[18F]FDG-PET/CT versus other imaging

Comparison of extended vascular US and 2-[18F]FDG-PET/CT showed comparable diagnostic accuracy in a cohort of suspected GCA (53). However, US was more sensible for temporal arteries vasculitis and popliteal vasculitis and 2-[18F]FDG-PET/CT was more performant for thoracic and abdominal aorta vasculitis. Thus, these two imaging modalities may be complementary. The advantages of US over 2-[18F]FDG-PET/CT are its availability, the absence of irradiation and a lower-cost imaging. However, it is operator dependent and does not detect alternate diagnosis such as neoplasia.

Multiple studies have shown comparable diagnostic accuracy between CT angiography (CTA) and PET/CT (25, 54–56). A higher correlation of PET with inflammatory markers was found (25, 55). The main advantages of CT over PET alone were the better evaluation of parietal damage and its availability. However, combination of PET with CT allows better evaluation of parietal damage even if reconstructed slice thickness remains superior to CT alone (∼3.5 mm vs. ∼2 mm) (Figure 1E).

In a prospective study comparing early diagnosis performance of MRI and 2-[18F]FDG-PET/CT, their diagnosis accuracy were comparable, however, 2-[18F]FDG-PET/CT detected more vascular regions involved than MRI (57). It should be noted that both are poorly correlate with clinical disease activity in patients with preexisting immunosuppressive therapy (48, 58). We summarize diagnostic performances of different imaging modalities for baseline evaluation in Table 2.

**TABLE 2:** Study characteristics and main findings on the diagnostic accuracy by angiography, ultrasound, CTA, magnetic resonance angiography (MRA) and 18-fluorodeoxyglucose positron emission tomography/computed tomography (2-[18F]FDG-PET/CT) at baseline in giant cell arteritis (GCA) and Takayasu arteritis.

	Angiography	US	CTA	MRA	2-[18F]FDG-PET/CT
Stenosis	+++	+++	+++	++	-
Artery wall thickness	-	+++	+++	++	-
Aneurysm	+++	+++	+++	+++	-
Parietal inflammation	-	+	++	++	+++
Flow	+	+++	-	+++	-
GCA							
	**References**	**Design**	**Population**	**Reference standard**	**Index test**	**Performance**	**Risk of bias based on EULAR evaluation (66)**
US	Luqmani et al. (14)	Prospective	381	Clinical diagnosis at 6 months (6m) or positive TAB	Halo/stenosis/occlusion (cranial arteries)	Se 54%, Sp 81% PPV 73% NPV 69%	Moderate
	Rinagel et al. (80)	Meta analysis	1,062 (20 studies)	Positive TAB	Halo/stenosis/occlusion (cranial arteries)	Se 78% Sp 79% PLR 3.80 NLR 0.29	Moderate
	Nielsen et al. (81)	Prospective	46	Clinical diagnosis and positive PET	Halo/compression sign (cranial and extra cranial arteries)	Se 97% Sp 100%	Low
	Hop et al. (82)	Retrospective	113	Clinical diagnosis 6 months	Halo/occlusion (cranial and extra cranial arteries)	Se 71% Sp 93%	Low
	Skoog et al. (83)	Restrospective	201	Clinical diagnosis at 6 months	Halo/compression sign (cranial and extra cranial arteries)	Se 95% Sp 98%	Moderate
CTA	Lariviere et al. (25)	Prospective	24	Clinical diagnosis at 6 months	Wall thickening+contrast enhancement score (1–4)	Se 73% Sp 84% PPV 84 NPV 64%	Low
MRA (cranial arteries)	Bley et al. (84)	Prospective	32	ACR criteria or positive TAB	Wall thickening+contrast enhancement score (0–3)	Se 80.6% Sp 97%	Low
	Siemonsen et al. (80)	Retrospective	28	ACR criteria or positive TAB	Wall thickening+contrast enhancement score (0–3)	Se 80% Sp 80%	Moderate
	Rhéaume et al. (85)	Prospective	171	ACR criteria or positive TAB	Wall thickening+contrast enhancement score (0–3)	Se 93.6% Sp 77.9% PPV 48.3% NPV 98.2%	Moderate
PET/CT	Blockmans et al. (21)	Retrospective	69	Clinical criteria and positive TAB	Visual intensity of FDG uptake	Se 56% Sp 98% PPV 93% NPV 80%	Moderate
	Soussan et al. (24)	Meta analysis	127 (8 studies)	ACR criteria or positive TAB	Visual or semiquantitative analysis of FDG uptake	Se 90% Sp 98% PLR 28.7 NLR 0.15	Moderate
	Lariviere et al. (25)	Prospective	24	Positive TAB	Visual intensity of FDG uptake	Se 66% Sp 100% PPV 100% NPV 64%	Low
	Sammel et al. (28)	Prospective	64	Positive TAB	Visual intensity of FDG uptake	Se 92% Sp 85% PPV 61% NPV 98% AUC 88%	Low
**TA**							
	**References**	**Design**	**Population**	**Reference standard**	**Index test**	**Performance**	**Risk of bias based on EULAR evaluation (66)**
US	Barra et al. (72)	Meta analysis	63	ACR Criteria and/or angiography	Carotid Intima-media thickness >1 mm	Se 81% Sp 100%	Moderate
CTA	Yamada et al. (81)	Retrospective	25	Conventional angiography	Luminal changes: stenosis, occlusion, dilatation	Se 67% Sp 100%	Low
MRA	Kumar et al. (86)	Retrospective	16	Conventional angiography	Luminal changes: stenosis, occlusion, dilatation	Se 91% Sp 88%	High
	Yamada et al. (87)	Retrospective	30	Conventional angiography	Luminal changes: stenosis, occlusion, dilatation	Se 100% Sp 100%	Low
	Barra et al. (72)	Meta analysis	182	Conventional angiography	Luminal changes: stenosis, occlusion, dilatation	Se 92% Sp 92%	Moderate
PET/CT	Santhosh et al. (65)	Retrospective	51	ACR criteria	Intensity of FDG uptake	Se 83% Sp 90%	Moderate. Evaluated all together performance for both diagnosis and disease activity

Se, sensibility; Sp, specificity; PPV, positive predictive value; NPV, negative predictive value; PLR, positive likehood ratio; NLR, negative likehood ratio; AUC, area under the curve; TAB, temporal artery biopsy.

### Conclusion 2-[18F]FDG-PET/CT and GCA

To sum up, 2-[18F]FDG-PET/CT is a useful diagnosis to assess diagnosis and prognosis of GCA.

It can be used in two situations: first, GCA is confirmed or highly probable, for example a high pretest probability and positive US or TAB. In this clinical situation the role of 2-[18F]FDG-PET/CT is to early detect large vessel involvement and to predict vessel damage, particularly thoracic aortitis which is associated with an increased risk of developing thoracic aorta dilatation (42, 44).

Secondly, GCA is suspected but uncertain. For example in patients presenting with constitutional symptoms, FUO, suspected LV involvement or signs of PMR with an intermediate or low pretest probability. In this clinical situation, 2-[18F]FDG-PET/CT is useful to detect signs of vasculitis and search for an alternate diagnosis: signs of PMR, neoplasia, other inflammatory diseases (sarcoidosis) or infection.

Its role in the follow-up of patients with GCA is not well-established. We propose to use 2-[18F]FDG-PET/CT during follow-up of GCA patients depending on clinical and biological parameters evolution to aid in therapeutic decisions: If patient present with clinical symptoms (extracephalic) but without inflammatory markers, a negative TEP may help in deciding to stop or not restart treatment. Also, in a patient with increased biological markers without clinical signs, a positive TEP may detect preclinical lesions and help in deciding to restart or increase anti-inflammatory treatment.

### 2-[18F]FDG-PET/CT and takayasu arteritis

Takayasu arteritis (TA) is the second primitive vasculitis affecting predominantly large vessels (1). It is ubiquitous but the highest incidence is found in Asia (59). Contrary to GCA it affects mainly patients under 40 years, has a higher F/M sex ratio and differs by clinical presentation and disease course (60).

### 2-[18F]FDG-PET/CT and TA diagnosis

There is no gold standard for diagnosis of TA and artery biopsy is not routinely available. Diagnosis is mainly based on the presence of characteristic imaging of large arteries in young patients under 50 years with clinical signs and/or elevated inflammatory markers (61).

Patients with TA may present with vascular symptoms attributable to arteritis but also systemic symptoms or “non-vascular” symptoms. Systemic symptoms may precede the vascular phase and are non-specific. They encompass fever, skin manifestations, arthralgia, episcleritis. Also, TA may be associated with other inflammatory diseases, such as sarcoidosis, spondylarthritis, or Crohn disease (62).

TA predominantly affect subclavian and common carotid arteries but aorta and all its branches may be involved (60). The disease is often diagnosed during the vascular phase which results from vascular complications: stenosis in >90% of cases, aneurysm in 20% of cases (63).

Appropriate imaging is the mainstay for the diagnosis of TA (Table 2). Based on its performance to investigate mural inflammation and/or luminal changes and the young age of the patients, European guidelines recommend angio-MRI as the first line imaging option replacing angiography (2). Moreover, to assess peripheral artery disease, French guidelines propose vascular doppler ultrasound to evaluate vessel wall morphology and blood flow (61).

We did not find study evaluating the accuracy of 2-[18F]FDG-PET/CT as a diagnostic tool only in TA. However, based on current clinical practice, recent 2022 ACR/EULAR classification criteria for Takayasu arteritis fully integrate evidence of vasculitis in the aorta or branch arteries confirmed by vascular imaging: CT/catheter-based/magnetic resonance angiography (MRA), ultrasound and PET (58, 64). One study by Santhosh et al. (65) studied 2-[18F]FDG-PET/CT as diagnostic tool but also included activity evaluation. Other studies or meta-analysis focused on 2-[18F]FDG-PET/CT as a measurement of the disease activity or included both GCA and TA. Similarly, there was no study on 2-[18F]FDG-PET/CT as diagnostic tool in TA included in the meta-analysis informing the EULAR guidelines on imaging (66).

### 2-[18F]FDG-PET/CT and TA prognosis

In a multicentric retrospective study of TA patients, relapse were observed in 43% of patients and vascular complications occurred in 38% of patients after a median follow up of 6.1 years (67). Main vascular complications in TA are: neurovascular disease (stroke, transitory ischemic attack), ischemic retinopathy, cardiovascular complications ranging from aortic regurgitation to pulmonary hypertension including coronaropathy and microvascular ischemia, renovascular disease, and peripheral artery disease. Risk factors for relapse were male sex, high CRP and carotidynia at diagnosis. Progressive disease, thoracic aorta involvement and retinopathy were associated with vascular complications (67).

One retrospective study evaluated the predictive value of 2-[18F]FDG-PET/CT in 32 patients with baseline 2-[18F]FDG-PET/CT and a median follow up of 83.5 months. Maximal standardized uptake value (SUVmax) in arteries ≥1.3 seemed to be associated with disease relapse [Odds ratio (OR): 5.667; 95% confidence interval (95 CI): (1.067–30.085)] and the need to change therapy [OR: 7.933; 95 CI: (1.478–42.581)]. Interpretation of these results must be cautious because of potential bias due to study design and very large 95% confidence interval of ORs. Also, there was no association between SUVmax intensity at baseline and the development of ischemic events, new angiographic lesions or sustained remission (68). In a recent prospective cohort to assess whether vascular 2-[18F]FDG-PET/CT activity is associated with angiographic change in LVV including 38 TA patients, lack of 2-[18F]FDG-PET/CT activity was strongly associated with stable angiographic disease, *P* < 0.01 (44). An arterial territory with baseline 2-[18F]FDG-PET/CT activity had 20 times increased odds for angiographic change compared to a paired arterial territory without PET activity. Angiographic progression with arterial damage was preceded by the presence of 2-[18F]FDG-PET/CT activity (44).

### 2-[18F]FDG-PET/CT and monitoring TA activity

Treatment of TA is based on glucocorticoids often associated with methotrexate or anti-TNFα in severe disease or in case of steroids dependence (15, 45, 61). There are no consensual criteria for assessing TA activity. Inflammatory markers are poorly correlated with angiographic progression and may remain negative in 30% of patients with clinically active disease (69). Two tools are commonly used : First the NIH criteria and more recently, the ITAS2010 criteria which is increasingly being used (70, 71).

A meta-analysis including 131 patients with TA evaluated 2-[18F]FDG-PET/CT performance for assessing activity of disease based on NIH and showed a sensitivity and specificity of 84% (24). All four included studies had a retrospective design. These results were confirmed in a second meta-analysis including 57 studies, mainly cross-sectional and of low methodological quality. The pooled sensitivity was 81% and specificity 74% (72). A recent longitudinal study included 126 patients with LVV (GCA = 50; TAK = 76) with 2-[18F]FDG-PET/CT at enrollment and follow up. Global arterial FDG uptake was quantified with PETVAS. After a median follow up of 2.6 years, there was no significant decrease in PETVAS in TA patients. Also, there was no direct correlation between PETVAS during follow up and clinical and biological activity (73).

One case report suggested that 2-[18F]FDG-PET/CT may not detect pulmonary artery (PA) involvement in TA) (74). This was infirmed in a study Gao et al. (75) which compared performance of 2-[18F]FDG-PET/CT versus CTPA or AMR in TA patients with PA involvement. 2-[18F]FDG-PET/CT was as sensible as radiological imaging (71.4 vs. 92.9%, *P* = *0.250*) and seemed to have higher specificity (91.7 vs. 37.5%, *P* = *0.001*).

Finally, a multimodal assessment of TA activity was proposed by amalgamating the sum of mean SUV, ESR and soluble interleukin-2 receptor (IL-2Rs) which seemed concordant with NIH and ITAS2010 criteria (76). However, the population included had different disease course and treatment. This model needs further validation using prospective studies and homogenous population.

### Conclusion PET/CT and TA

The place of 2-[18F]FDG-PET/CT in TA management remains poorly defined. Diagnosis and disease activity assessment in TA can be challenging as patients may not have overt clinical symptoms or elevated CRP at diagnosis or during periods of active disease. Combination of non-invasive vascular imaging such as doppler ultrasound, MRA, CTA, and 2-[18F]FDG-PET/CT remains the first line modality for diagnosis of TA and is essential to monitor vascular disease in patients with TA. During follow up, new areas of arterial damage can develop despite apparent clinical and biological remission in TA. 2-[18F]FDG-PET/CT cannot be systematically recommended for follow up but incorporate the use of 2-[18F]FDG-PET/CT with non-invasive vascular imaging may complete multimodal imaging assessment of disease activity and risk of vascular damage.

### Prospects

Novel PET imaging techniques are progressively used or under research.

Positron emission tomography/MRI has been evaluated in large vessel vasculitis and allow analysis of different pattern: fibrous vs. inflammatory pattern (77). Its place in LVV, TA particularly, remains to be specified.

Van der Geest et al. (49) recently reviewed novel PET imaging techniques using novel cell targets and novel tracers. These techniques could improve imaging accuracy by using a more specific cell uptake of FDG with less background activity. Also, it could enhance activity evaluation after treatment (78). Some of these novel targets are: T cells targeted radio tracers (IL2-R, CD4, CD8), macrophages [Translocator protein (TSPO), mannose receptor (CD206), folate receptor and others], B cells, activated fibroblasts (Fibroblast activation protein alpha pathway), endothelial cells (VCAM-1).

Some drawbacks have been underlined by Van der Geest et al. (49): the risk of high irradiation dose, the complexity and cost of radiotracers development and the need of clinical study to confirm their utility.

## Conclusion

This review illustrates that 2-[18F]FDG-PET/CT is a powerful metabolic imaging tool that may help improving early diagnosis, current classification, and prognostic assessment in LVV. In patients with a clinical suspicion for LVV, 2-[18F]FDG-PET/CT can help to diagnose LVV especially at the early onset of disease or in case of non-specific signs. Early recognition of LVV is essential because irreversible ischemic complication (e.g., stroke, vision loss, myocardial infarction) almost always occur early, prior to steroids therapy. Moreover, the presence of vascular 2-[18F]FDG-PET/CT activity can precede angiographic change and progression in LVV. Conversely, the disappearance of 2-[18F]FDG-PET/CT uptake after effective therapy is possible. Thus, 2-[18F]FDG-PET/CT may be useful to evaluate treatment efficiency as well as for detection of LVV relapse and vascular complication at an early stage. Persistent activity however, is difficult to interpret, and its impact on disease treatment modifications is not well-known yet and needs further research. 2-[18F]FDG-PET/CT may help clinician to determine patients with more active, diffuse, and severe LVV requiring a more intensive treatment and close monitoring.

## Author contributions

KN and CC collected the data and wrote the manuscript. AV, VB, KC, RB, SM, and DS made imaging analysis. KN and CC were responsible for verification of all the underlying data and took full responsibility for the integrity of the work. All authors critically reviewed and approved the final version of the manuscript.
